# Validation and Test-Retest Reliability of New Thermographic Technique Called Thermovision Technique of Dry Needling for Gluteus Minimus Trigger Points in Sciatica Subjects and TrPs-Negative Healthy Volunteers

**DOI:** 10.1155/2015/546497

**Published:** 2015-06-07

**Authors:** Elżbieta Skorupska, Michał Rychlik, Włodzimierz Samborski

**Affiliations:** ^1^Department of Rheumatology and Rehabilitation, Poznan University of Medical Sciences, Fredry 10, 61-701 Poznan, Poland; ^2^Department of Virtual Engineering, Poznan University of Technology, Plac Marii Skłodowskiej-Curie 5, 60-965 Poznan, Poland

## Abstract

The aim of this study was to assess the validity and test-retest reliability of Thermovision Technique of Dry Needling (TTDN) for the gluteus minimus muscle. TTDN is a new thermography approach used to support trigger points (TrPs) diagnostic criteria by presence of short-term vasomotor reactions occurring in the area where TrPs refer pain. *Method*. Thirty chronic sciatica patients (*n=15* TrP-positive and *n=15* TrPs-negative) and 15 healthy volunteers were evaluated by TTDN three times during two consecutive days based on TrPs of the gluteus minimus muscle confirmed additionally by referred pain presence. TTDN employs average temperature (*T*_avr_), maximum temperature (*T*_max_), low/high isothermal-area, and autonomic referred pain phenomenon (AURP) that reflects vasodilatation/vasoconstriction. Validity and test-retest reliability were assessed concurrently. *Results*. Two components of TTDN validity and reliability, *T*_avr_ and AURP, had almost perfect agreement according to *κ* (e.g., thigh: 0.880 and 0.938; calf: 0.902 and 0.956, resp.). The sensitivity for *T*_avr_, *T*_max_, AURP, and high isothermal-area was 100% for everyone, but specificity of 100% was for *T*_avr_ and AURP only. *Conclusion*. TTDN is a valid and reliable method for *T*_avr_ and AURP measurement to support TrPs diagnostic criteria for the gluteus minimus muscle when digitally evoked referred pain pattern is present.

## 1. Introduction

The main problem of the pain research filed is the difficulty with an objective quantification of pain. Most authorities agree that a fair amount of pain is left undertreated, especially in the chronic form [1]. It has been estimated that in around 30% of patients consulting for pain in primary care the coexistence of myofascial pain syndrome (MPS) caused by trigger points (TrPs) was confirmed [2]. Unfortunately, this MPS is drug-resistant and very often diagnostically overlooked. Nevertheless, a grown interest in MPS has been lastly observed and the main controversy around pain studies, namely, an objective confirmation of TrPs presence, is again the main research theme. Two new methods, sonoelastography and magnetic resonance elastography, have been recently introduced allowing noninvasive imaging of TrPs [3, 4]. Both are not cheap or easily accessible; thus, TrP confirmation is still based on palpatory diagnostic criteria defined by Travell and Simons [5, 6].

Interestingly, for years MPS has been defined as nociceptive pain, but today the importance of sympathetic nervous system (SNS) activity for MPS pain propagation is indicated more and more frequently [7–9]. Vasomotor responses and hyperalgesia observed in the area of TrPs related referred pain can be also attributed to possible sympathetic mechanism [10–14]. This supports the last point of Simons' integrated hypothesis concerning TrPs etiology which states that autonomic modulation has a potential influence on the increase of ACh release, which can aggravate symptoms caused by TrPs [15]. It is commonly accepted that infrared thermovision (IRT) camera can objectively support the diagnosis of pain patients, especially when SNS activity is involved [16]. High IRT reliability for muscle examination [17, 18] and significant correlation between pressure pain threshold and temperature differences in MPS have been recently proved [19]. Moreover, a new idea for pain medicine studies is a stress loading IRT test, for example, by cold/warm stress, exercise, pharmacological stress, vibration, and visual stimulation. Enhanced sensitivity and specificity for the diagnosis of SNS related diseases, for example, CRPS, by cold stress thermography has been recommended. Unfortunately, it causes pain in the patient, and no standardized guidelines for the stress loading test have been established [20–22]. Additionally, some authors claim that IRT with its temperature measurement and visual thermogram analysis is not sufficient as a diagnostic tool in medicine as it needs to provide thermogram analysis more objectively [16]. Interestingly, the new awarded method called Thermovision Technique of Dry Needling (TTDN) allows measuring changes of isothermal-area size (every thermogram can be divided into subareas of 0.7°C each) [23]. Additionally, for TrPs-positive subjects TTDN can measure intensive short-term vasomotor response related to noxious stimulation of TrPs alone. If every TrP can provoke vasomotor response, TTDN can objectively support Travell and Simons' TrPs diagnostic criteria.

For the purposes of the study, trigger points of the gluteus minimus muscle were considered because of the most intensive and longest referred pain pattern which spreads almost in the whole lower limb [5, 6]. Thus, this muscle seems to be the best choice for TTDN validation conducted for the first time. According to the literature, trigger points within the gluteus minimus muscle can be observed among radicular sciatica patients as secondary symptoms due to disc lesion or can provoke sciatic-like pain, pseudoradicular pain [26, 27]. Objective distinction of radicular and nonradicular sciatic pain is not possible. Based on the literature, a patient who presents, for example, the lack of positive Lasegue test result accompanied by the lack of neurological signs of sciatic nerve irritation should be diagnosed as pseudosciatica or sciatic-like pain. However, Rolke posited that pseudoradicular and radicular low back pain should be considered to be disease continuum rather than different entities [28].

The coexistence of gluteus minimus TrPs in around 30% of subacute and chronic radicular sciatica patients was proved [10, 11], as well as the presence of active TrPs among sciatica-like cases [12, 13]. Based on some previous studies, it can be hypothesized that the presence of short-term vasomotor changes in the area where TrPs provoke pain may enable objective trigger points confirmation. Whether the commonly used palpatory criteria are reliable is controversial. Thus, it is very important to find an objective and easy tool for TrPs confirmation.

The main aim of this study was to validate Thermovision Technique of Dry Needling (TTDN) and examine its test-retest reliability for the gluteus minimus muscle. The additional aim was to analyze the average value of TTDN components in the light of clinical division.

## 2. Material and Method

The study was conducted in accordance with the Declaration of Helsinki approved by the Ethics Committee of Poznan University of Medical Sciences (number 772/14). It was prospectively registered at the Australian New Zealand Clinical Trials Registry (ACTRN12614001168640). All subjects gave written informed consent to participate in the study before data collection. A detailed description of all examinations and treatment procedures, including dry needling (DN), as well as of risks involved in the study was provided to the participants. Participants had the right to refuse the DN treatment and withdraw from the study at any time without penalty.

### 2.1. Subjects

Thirty Caucasian chronic sciatica subjects, where half of them were TrPs-positive and half TrPs-negative, and fifteen Caucasian healthy volunteers were recruited to the study from Poznan GP doctors, the University Pain Clinic, by press announcement and University staff. The age ranged from 35 to 58 years (average 46.6 ± 8.7 y). Sciatica subjects were diagnosed by an experienced neurologist towards radicular origin of sciatica on the basis of bedside examination, extensive neurological screening examination accompanied by a positive straight leg test, and magnetic resonance imaging results.

*Clinical Criteria for Gluteus Minimus Muscle TrPs Confirmation*. According to Travell and Simons, the taut band (one of the essential criteria) is unlikely to be palpated because it lies deeper than both the gluteus maximus and the gluteus medius muscles. However, TrPs spot tenderness can be clearly localized. Additionally, the referred pain pattern is more likely to be observed when needle encounters TrPs rather than when sustained pressure on the tender spot is applied [5, 6].

The diagnosis of TrPs within the gluteus minimus muscle in the present study was based on Travell and Simons' clinical criteria [5, 6]. However, due to the lack of the taut band of the gluteus minimus muscle the presence of the confirmatory sign (referred pain pattern) was added to the full range of possible essential criteria for strong evidence. Active trigger points within gluteus minimus were confirmed if spot tenderness, pain recognition, and limited range of movement were confirmed and the full referred pain pattern felt in the thigh and calf was present (evoked by snapping palpation).

*Key Inclusion Criteria*. Key inclusion criteria for sciatica patients were as follows: age between 30 and 60 (inclusive), both lower limbs present, pain duration >3 months, >3 on the 1–10-point VAS scale of leg pain, with this being the dominant pain problem and pain felt minimum to the calf, and the results of the straight leg test between 30 and 60 degrees.

Key inclusion criteria for healthy volunteers were general good health condition, age between 30 and 60 (inclusive), both lower limbs present, and lack of latent trigger points within the gluteus minimus muscle.

*Key Exclusion Criteria*. Subjects were excluded owing to complex regional pain syndrome, cauda equina syndrome, previous back surgery, spinal tumors, scoliosis, pregnancy, coagulant treatment, disseminated intravascular coagulation, diabetes, epilepsy, infection, inflammatory rheumatologic diseases, stroke, or oncological history.

### 2.2. Methods

All patients recruited to the study were consecutively examined using TTDN (detailed description below). Also side-to-side IRT comparison of the lower limbs was performed.

The IRT camera operator was not aware of the results of gluteus minimus TrPs examination, and the physician who performed dry needling was not aware of the IRT results during the procedure. TTDN was performed three times during two consecutive days. On the first day, TTDN was performed once and, on the second, two sessions with one-hour break were performed for the same marked points. A thermovision touchless camera (NEC-AVIO TVS-200EX) with a 8–14 *μ*m wave band, temperature resolution better than 0.080°C, and sensitivity of 80 mK and working in real time was applied. The camera was equipped with a high-speed (60 Hz) uncooled FPA 320 × 240 (*H* × *V*) pixels VOx (vanadium oxide) microbolometer. For thermal images analysis, the specialist program “Thermography Studio 2007 Professional” was used. Every day before the procedure every participant was reexamined towards active gluteus minimus TrPs. Then, the localization of the two most active TrPs was marked. For non-TrPs sciatica subjects and healthy volunteers, two nontender points were marked. The dry needling specialist had no knowledge of whether the marked points were TrPs or non-TrPs.

#### 2.2.1. General Protocol for TTDN

TTDN result is thought to be positive if significant *T*_sk_ (maximum and average temperature) changes accompanied by significant isothermal-area changes in the area of pain are confirmed. Isothermal-area is defined as an area of the patient's body with the same temperature at the same moment of time. The TTDN method was partially described in the authors' previous studies [10, 11].

*(a) Statement*. Thermographic images were recorded by an expert following a standard protocol recommended by the Academy of Neuromuscular Thermography. The expert also evaluated the images [24]. Patients were instructed to avoid physiotherapy and manual therapy 24 hours prior to the test and to avoid using nasal decongestants, analgesics, anti-inflammatory drugs, or any substances affecting the sympathetic function. They were also instructed not to drink coffee or alcohol and to refrain from smoking 2 hours before the recording.

To obtain the stability of the patient's body temperature and to ensure the adjustment of the recording camera's temperature to the interior conditions, the evaluation began 30 minutes after the patient had entered the examination room. Thermal isolation of the evaluated area from other thermal factors that might have influenced the evaluation, including other parts of the patient's and doctor's bodies, was ensured. Moreover, when performing thermovision imaging, the general rules of camera usage were followed.

*(b) Patient Preparation. *Consider the following:

1. Drawing pain on the pain diagram.
2. Side-to-side comparison of the painful area by IRT was as follows:
  (a) Comparison of *T*_sk_ differences in the painful leg versus the opposite side.
  (b) When a *T*_sk_ decrease of more than 0.5°C in the painful area compared to nonpainful leg was observed, then the feature of neuropathic pain was considered possible.
3. Positioning the patient according to dry needling rules for the examined muscle. In this position, thermovision images of the patient were recorded. For adequate representation of dimensions, a calibration standard was applied. The next step involved recording the “base” image. The image was recorded to establish the initial level of the patient's temperature parameters.
4. When the above-mentioned conditions were met, dry needling under IRT control was performed.

*(c) Dry Needling (DN) Session under IRT Control*. Dry needling of every point lasted for 5 minutes. During the whole procedure, the subarea of referred pain reported by the patient was recorded. After the needling of both marked points was completed, further thermovision imaging was performed. The IRT observation lasted for six consecutive minutes after DN. At the end of the procedure, the patients were asked to answer the question: “Was the pain evoked by needling similar to your daily pain?”

*(d) Thermogram Analysis*

*Skin Temperature Changes*. The analysis of thermograms assumed skin temperature changes: maximum temperature (*T*_max_), minimum temperature (*T*_min_), and average temperature (*T*_avr_) in the observed area after the dry needling session and during 6 minutes after DN.

*Analysis of the Impact of Vasomotor Reactions Presence on the Referred Pain*. The expected vasomotor changes in the area of TrPs related referred pain were named autonomic referred pain (AuRP) if present (Table 1). Post-DN and postobservation analysis assumed the calculation (in cm^2^) of skin isothermal-area changes, with the reference point being *T*_max at rest_ for AURP-vasodilatation, and *T*_min at rest_ for AURP-vasoconstriction from the thermogram at rest. Additionally, the size of high *T*_sk_ isothermal-area (1.5°C below *T*_max at rest_) and low *T*_sk_ isothermal-area (1.5°C above *T*_min at rest_) was calculated. The size of each isothermal-area was recalculated from cm^2^ to the percentage value.

*TTDN for Gluteus Minimus Trigger Points—Additional Information*. The area to be observed by IRT was chosen according to the gluteus minimus referred pain pattern. The examined patients were positioned on the side, on the uninvolved extremity with the hip and knee flexed. The muscle was needled with flat palpation perpendicular to the muscle along the counter of the iliac crest. Strong depression of the subcutaneous tissue was applied in order to reduce the distance of the skin from the muscle. Depth of penetration depended on the amount of adipose tissue [25]. Therapeutic needling was performed with 0.30 mm diameter, 60 mm long sterile acupuncture needles SE L (Serin Corp., Shizuoka, Japan). Each needle was packed separately.

### 2.3. Statistical Analysis

*TTDN Results Dependent on Clinical Division*. The chronic sciatica subjects and healthy volunteers were compared, with trigger points coexistence being the differentiating criterion. For the strong evidence of data presented, the significance level was set based on exact tests, not on the default asymptotic method. Exact two-way Mann-Whitney *U* tests were performed in order to ensure that data were representative of the whole population of possible data values. Tests were applied to compare the differences for maximum, minimum, and average skin temperatures and the percentage size of isothermal-area for the state after dry needling and, secondly, for the postobservation state. Values, figures, and tables in the text were expressed as ± standard error of the mean (SE). Significance level was set at *p* < 0.05. IBM SPSS Statistics, version 20′′, was used.

*Validity and Reliability of TTDN*. A validity analysis was performed to confirm the validity of TTDN towards sensitivity and specificity of the obtained results (skin temperature changes and percentage size of vasomotor responses). The components of validity that were used in this study include sensitivity and specificity, as well as positive and negative predictive values. Tables 2 and 3 provide definitions and explanations for the components of validity [30, 31].

*Reliability of the TTDN Method*. Test-retest was used to check the reliability of TTDN according to the Guidelines for Evaluating and Expressing the Uncertainty of NIST Measurement Results. TTDN was performed using the same experimental tools, the same observer, the same measuring instrument used under the same conditions, the same location, and repetition over a short period of time on the same patient. Intraclass correlation coefficients (ICC) for test-retest reliability (intraobserved variability) were calculated to illustrate the differences between repeated measures [29]. Intraclass correlation coefficients above 0.90 were considered excellent, values form 0.75 to 0.90 were considered good, and below 0.75 considered poor to moderate [32].

To overcome the problem of tests agreement, the *κ* coefficient was used. The guidelines by Landis and Koch were used to interpret the obtained *κ* values and are presented in Table 4 [33].

## 3. Results

### 3.1. TTDN Results Dependent on Clinical Division

Among the sciatica group, only two patients (both TrPs-positive subjects) felt pain going to the foot as a daily complaint. During TTDN, dry needling related pain sensation consistent with gluteus minimus referred pain was confirmed for TrPs-positive exclusively. Among TrPs-positive sciatica subjects, DN related pain sensation on the thigh during TTDN was confirmed for every subject in all three sessions. DN reactivity for the calf for the three sessions was 100%, 80%, and 73.3%, respectively. Two TrPs-positive subjects complained of the daily pain of the foot, but during the procedure they did not report needle sensation going to the foot.

*Skin Temperature Changes Related to TTDN*. TTDN confirmed *T*_sk_ and isothermal-area changes for every subject. The exact two-way Mann-Whitney *U* tests confirmed significant increase of *T*_max(thigh,calf,foot)_ and *T*_avr(thigh,calf)_ for TrPs-positive sciatic group as compared to TrPs-negative sciatic patients and heathy volunteers (all *p* < 0.05) (Tables 5 and 6). The sciatic group presented contrary *T*_sk_ changes dependent on TrPs and DN related referred pain presence. For TrPs-negative, *T*_sk_ decrease contrary to *T*_sk_ increase of TrPs-positive was observed.

*Isothermal-Area Changes Related to TTDN*. None of TrPs-positive subjects presented vasoconstriction or any significant changes of the low *T*_sk_ isothermal-area and *T*_sk_ decrease. Significant *T*_sk_ and isothermal-area changes for every TrPs-positive sciatica subject were confirmed.

**Figure 1:**
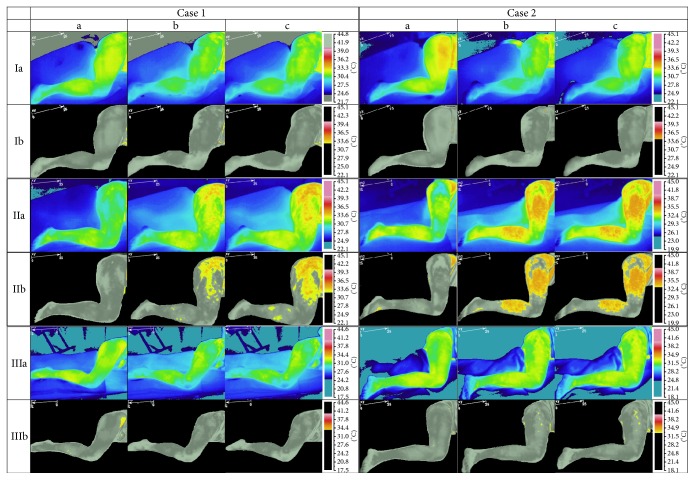
TTDN related thermograms presenting temperature reaction measured on skin surface of TrPs and non-TrPs sciatica patients and healthy volunteers. In rows: I: two cases of TrPs-negative sciatica, II: two cases of TrPs-positive sciatica, and III: two cases of healthy volunteers were shown. In rows Ia, IIa, and IIIa full thermogram was presented. In rows Ib, IIb, and IIIb the picture of the isothermal-area above *T*_max at rest_ called AURP was shown (visualization of the vasodilation). In columns: (a) Before TTDN (initial state), (b) state immediately after DN, and (c) state immediately after observation were presented.

The exact two-way Mann-Whitney *U* tests confirmed a significant increase of the observed isothermal-area (calculated together: AURP and high *T*_sk_ isothermal-area) for TrPs-positive sciatica subjects compared to TrPs-negative sciatica patients and healthy volunteers (all *p* < 0.05) for both post-DN and postobservation phases (every *p* < 0.05). After dividing the isothermal-area into high *T*_sk_ isothermal-area and AURP (shown in Figure 1), the results were as follows.

The exact two-way Mann-Whitney *U* tests confirmed a significant increase of the high *T*_sk_ isothermal-area (thigh and calf) for TrPs-positive compared to TrPs-negative sciatica subjects (*p* < 0.05) but not to healthy volunteers (Table 7). The significant increase of AURP (vasodilatation) for TrPs-positive compared to TrPs-negative and healthy volunteers was confirmed (The exact two-way Mann-Whitney *U* tests; every *p* < 0.05). The average value of the percentage increase of AURP was shown in Table 8.

### 3.2. Validity and Reliability of TTDN

There were considerable differences detected in the results of TTDN when it was performed on the non-TrPs sciatic group, TrP-positive sciatic group, and non-TrPs healthy volunteers. The agreement between TTDN and TrPs diagnosis was almost perfect (according to *κ*) for changes of *T*_avr_ and isothermal-area above *T*_max at rest_ of the thigh and calf (Tables 9 and 10). In terms of validity components, TTDN was useful for identifying the positive values of *T*_max_ changes and isothermal-area (below *T*_max at rest_) or full AURP (namely, below and above *T*_max at rest_ calculated together) changes but was not useful for identifying the negative values (specificity). Although *T*_max_ and isothermal-area below *T*_max at rest_ or full AURP have high sensitivity, the low specificity does not allow recommending their diagnostic value.

## 4. Discussion

The results of skin temperature (both *T*_avr_ and *T*_max_) and isothermal-area significantly differ for TrPs-positive sciatica subjects compared to non-TrPs sciatica subjects and healthy volunteers (*p* < 0.05; Tables 5–8), which is consistent with the previous study involving sciatica subjects only [10, 11]. However, the main purpose of the present study was to check the validity and reliability of TTDN. This new IRT method was assumed to allow observing vasomotor and temperature reactions related to TrPs and was validated for its ability to distinguish active TrPs from non-TrPs of the gluteus minimus muscle and non-TrPs of healthy volunteers. It was found that TTDN identifies active gluteus minimus TrPs for every positive subject. The most discriminatory indicators for TrPs presence were *T*_avr_ increase and the presence of high *T*_sk_ increase above *T*_max_ (isothermal-area defined as AURP; Tables 9 and 10) in the area where dry needling intensified pain. The results showed that TTDN validly measured *T*_avr_ changes and the presence of AURP (grey picture in Figure 1) for active TrPs of the gluteus minimus muscle. The ICC results confirmed moderate reliability for AURP presence and poor to moderate for *T*_avr_ increase. However, these two types of thermogram analysis showed almost prefect agreement according to *κ* (Tables 9 and 10), which is recommended as a more suitable measure of agreement among nonexchangeable observers in comparison with ICC. It seems that AURP and significant *T*_avr_ increase can objectively support Travell and Simons' clinical criteria for active TrPs within the gluteus minimus muscle when referred pain is evoked by snapping palpation [5, 6]. However, similar tendency for AURP and *T*_avr_ for some of the healthy volunteers was observed (AURP, not exceeding 2.5% with insignificant *T*_avr_ of +0.14°C) (Tables 6 and 8), and small changes can be explained by *T*_sk_ differences in time due to the physiological variability of blood flow [34].

IRT reliability was checked for the muscle examination and was found more reliable compared to the present study, but they examined the thermograms of the subjects at rest [17, 18]. The quantitative measurement of the percentage value of isothermal-area changes is presented for the first time and allowed observing the changes of a specific subarea of defined temperature very precisely. However, it should be underlined that it was possible because dry needling provoked intensive *T*_sk_ changes (Figure 1), which again is unusual compared to acupuncture studies where postneedling stimulation was limited to the site of needling [35, 36]. TTDN allowed observing dynamic thermogram changes due to the stimulation by dry needling and the fact that DN is a treatment technique for TrPs release [37]. The difference between the first and the last session could have resulted from the therapeutic DN effect, apart from the skin blood flow variability.

Moreover, it should be underlined that the strong inclusion and exclusion criteria for TrPs confirmation did not allow claiming that TTDN is a new objective method for every gluteus minimus TrP confirmation. It has been claimed that digitally evoked referred pain pattern from the gluteus minimus muscle is rare and for the diagnostic purposes the presence of a tender point and needle encouraged referred pain were postulated as diagnostic criteria for that muscle.

The diagnostic criteria established for the gluteus minimus muscle in the present study were as severe as possible because it has been lastly postulated that the methodological quality of the majority of studies conducted for the purpose of establishing trigger point reproducibility is generally poor [38]. Tough et al. [39] indicated that only 15% of authors used the combination of a tender spot in a taut band of a skeletal muscle, patients' pain recognition, predicted pain referral pattern, and local twitch response. Thanks to TrPs diagnostic criteria used in the present study, the diagnosis of TrPs presence cannot be questioned and, additionally, the consistency condition regarding the referred pain presence minimum to the calf ensures the group homogeneity. Of course, the choice of the gluteus minimus muscle for the first use of TTDN can be questioned. However, an advantage of this choice is the size of the referred pain pattern, which is one of the most extensive froms all of the TrPs referred pain patterns. Thus, it was predicted that the observed vasomotor reactions, if present, should be probably the widest and easily detectable by IRT.

*TTDN Results in the Light of the Previous Studies*. The use of thermography is not a new way for TrPs evaluation. Most of the authors tried to correlate the localization of TrPs with hot spots on the thermogram [16]. Brioschi et al. [40] analyzed most of the available studies on the subject and concluded that IRT findings represent an objective means of documenting TrPs if the thermogram is analyzed by an experienced physician skilled in clinical thermology together with clinical evaluation of the patient. However, in one of the oldest studies Swerdlow and Dieter [41] evaluated the sensitivity and specificity of medical thermography for the documentation of TrPs and they concluded that the localizations of TrPs and hot spots were not associated. Moreover, some other authors stated that skin temperature measurement in TrPs area cannot be used to detect myofascial tender spots [42]. Additionally, in the latest review regarding IRT application to TrPs, it has been stated that there are few studies evaluating the accuracy and reliability of infrared thermography for the diagnosis and assessment of TrPs. In the few studies present, there is no agreement on skin temperature patterns in the presence of TrPs [43].

IRT was also used for TrPs related referred pain area examination. Although Brioschi et al. [44] evaluated 304 chronic myofascial pain patients using IRT before and after DN or anesthetic infiltration, they found that referred areas were thermally asymmetric, and Kimura et al. [8] confirmed a significant decrease in *T*_sk_ over time after glutamate injection (nociceptive stimulation) in latent TrPs (*p* < 0.05), the usefulness of IRT for postnoxious stimulation of latent TrPs referred pain observation was contradicted by Zhang et al. [9]. On the other hand, one of the oldest studies on the subject presents results similar to the present study. Kruse Jr. and Christiansen [45] observed referred pain pattern by IRT when palpation pressure over TrPs lasted around 1 minute. Initially, they observed a small *T*_sk_ increase followed by a significant decrease in the area of the observed referred pain. The DN stimulation is a much more severe type of TrPs stimulation compared to pressure, and the time of stimulation in the present study was ten times longer. It can be hypothesized that a stronger noxious stimulation could provoke much more intensive *T*_sk_ increase and maybe Kruse Jr. and Christiansen [45] observed the same vasomotor reactions but of a weak and short form. Additionally, another difference between the studies is the localization of visible thermal response. They reported visible *T*_sk_ changes on the thermogram, more extensive than that of the reported referred pain during TrPs compression contrary to the present study, where AURP localization is limited precisely to the area where DN provoked pain during TTDN (Figure 1). The thermogram analysis before isothermal-area calculation was based on the indirect thermogram analysis, where the examiner had to distinguish subtle color differences. The isothermal-area calculation together with the thermogram of AURP (gray picture Figure 1) is a nonquestionable proof of significant vasomotor changes and can be easily interpreted by everyone.

Interestingly, the analysis of *T*_avr_ changes in the whole group of subjects in the present study (Table 6) clearly indicated *T*_avr_ decrease for non-TrPs, healthy volunteers, and needle insensitive foot of TrPs-positive sciatica subjects contrary to *T*_avr_ increase of DN reactive subarea of TrPs-positive subjects. Moreover, when the thermograms of non-TrPs sciatica subjects were observed some of them presented discreet vasoconstriction feature (Figure 1) and *T*_sk_ decrease (Tables 5 and 6). These results allow putting the question of whether the authors of the previous study who confirmed TrPs-related vasoconstriction really stimulated TrPs. On the other hand, maybe the type of TrPs (latent TrPs) or region of IRT observation (upper extremity) or type of noxious stimulation (glutamate injection) can explain contrary results [8, 9]. Further studies considering both active and latent TrPs, different regions of the body, and so forth are required.

*The New Idea—Isothermal-Area Calculation*. The standard usage of IRT in medicine is based on mean temperature and standard deviation within the fixed region of interest (ROi), as well as the visualized interpretation of the thermogram, where each temperature value (or a group of similar values) is attributed to a specified color. The color variation is the only indirect analysis and this type of interpretation is limited to the physician trained in thermology. Additionally, the use of spot temperature measurements or observation of selected values of *T*_max_ or *T*_avr_ may not produce the expected outcomes and the results obtained this way may differ a lot from the real situation. In summary, the comparison of *T*_avr_ of ROi is performed without considering the size of ROi. Thus, an error resulting from the statistical interpretation is possible. These facts can explain the controversies around the merit of IRT measurement in medicine. However, it has been postulated that considering the number of pixels in the observed ROi, that is, the qualitative evaluation of, for example, its size and shape, together with *T*_avr_ can lead to an objective interpretation of the results [16].

TTDN is the method which allows assessing the thermogram in this recommended way. The measurement of the size of isothermal-areas in the present study guarantees the precise results of the occurring process of thermal phenomena appearing on the human skin. In addition, TTDN analysis accounts for 100% of the information recorded on a single thermogram.

The next innovativeness of TTDN apart from calculating the size of ROi is the possibility of showing vasomotor reactions in TrPs referred pain area. The results are easy to interpret and not limited by a physician experience. The measurement of the size of isothermal-areas in the present study guarantees the precise results of the occurring process of thermal phenomena appearing on the human skin. In addition, the validity of TTDN confirmed the most discriminatory indicators for TrPs presence, *T*_avr_, and isothermal-area calculation.

## 5. Summary and Limitations of the Study

We are unaware of any other published studies regarding the validity and reliability of TrPs noxious stimulation by DN under IRT control to support the diagnosis of TrPs. The results of the present study were subject to the rigor and diagnostic protocol of the standards thermographic procedure. Moreover, side-to-side *T*_sk_ comparison of the patient at rest performed before the main procedure allowed excluding pain states related to sympathetic nervous system activity.

TTDN seems to be a promising tool for objective TrPs confirmation. However, this is the first study of this type and the validation of TTDN on other muscles with TrPs and some studies presenting a group with active and latent TrPs of the same muscle are required. After a series of studies that would give the same results, TTDN could be claimed as a new objective method for TrPs confirmation where DN is possible to perform.

The limitations of this study are the strong inclusion and exclusion criteria for the confirmation of gluteus minimus muscle TrPs. This muscle should be checked by TTDN when TrPs confirmation is based on tender point presence, which provoked typical referred pain when needle encountered TrPs.

Moreover, Travell and Simons claimed that autonomic phenomena (including vasomotor reaction) within TrPs referred pain area are limited to severe active TrPs only. In the present study, TrPs-positive sciatic patients can be assumed to probably have severe active TrPs due to the chronicity and length of the referred pain pattern evoked by snapping palpation. However, the severity of active TrPs still remains only a theoretical consideration because there are no criteria for distinguishing the severity of active TrPs [26]. Additionally, other TrPs, for example, within the upper trapezius muscle, should be examined by TTDN to answer the question if every TrPs present AURP is accompanied by *T*_avr_ increase.

Finally, the present study is limited by the lack of interrater reliability of TTDN.

## 6. Conclusion

TTDN is valid and reliable for *T*_avr_ and AURP measurement to support TrPs diagnostic criteria for the gluteus minimus muscle when a digitally evoked referred pain pattern is presented. In the light of clinical division, TTDN results indicate skin temperature increase for TrPs-positive contrary to decrease for TrPs-negative chronic sciatica patients and small changes (both increase/decrease) for healthy volunteers.

## Figures and Tables

**Table 1:** Isothermal-area analysis related to TTDN.

Initial state	After DN	After observation
*T*_min at rest_	If isothermal-area decreases below *T*_min at rest_—the confirmation of **AuRP (vasoconstriction)**	
	**Low ** **T**_**sk**_ ** isothermal-area (**1.5°C above *T*_min at rest_ **)**	

*T*_max at rest_	**High ** **T**_**sk**_ ** isothermal-area (**1.5°C below *T*_max at rest_ **)**	
	If isothermal-area increases above *T*_max at rest_—the confirmation of **AuRP (vasodilatation)**	

**Table 2:** Definitions of validity elements [24, 25].

Validity elements	Definition	Equation
Sensitivity	Proportion of the positive values that the test correctly identifies	*a* (*a* + *c*) × 100
Specificity	Proportion of the negative values that the test correctly identifies	*a* (*b* + *d*) × 100
Positive predictive value (PPV)	Proportion of the patients with positive test results who are correctly diagnosed	*a* (*a* + *b*) × 100
Negative predictive value (PNV)	Proportion of the patients with negative test results who are correctly diagnosed	*d* (*c* + *d*) × 100

**Table 3:** Validity elements [24, 25].

Test performed			
Results detected by the test	True picture		Total
	Patient has the condition	Patient is clear	
Positive test	A (true positive)	B (false positive)	A + B (total positive tests)
Negative test	C (false negative)	D (true negative)	C + D (total negative tests)
Total	Total subjects diagnosed with the condition	Total subjects without the condition	*N* (total number tested)

**Table 4:** Landis and Koch guidelines for *κ* interpretation [29].

*κ* value	Strength of agreement
<0.00	Poor agreement
0.00–0.20	Slight agreement
0.21–0.40	Fair agreement
0.41–0.60	Moderate agreement
0.61–0.80	Substantial agreement
0.81–1.00	Almost perfect agreement
1.00	Perfect agreement

**Table 5:** Mean value of maximum temperature changes.

*T*_max⁡_		Sciatica patients			Healthy volunteers
TTDN phases	Subarea	Non-TrPs&DN-negative	TrPs&DN-positive	*p*	Non-TrPs&DN-negative
After DN	Thigh	−0.12 ± 0.16	1.21 ± 0.18	*∗*/*∗∗*	0.9 ± 0.14
	Calf	−0.47 ± 0.15	0.66 ± 0.19	*∗*/*∗∗*	−0.05 ± 0.13
	Foot	−0.24 ± 0.14	0.1 ± 0.11	—	−0.05 ± 0.15

After observ.	Thigh	0.12 ± 0.18	1.29 ± 0.18	*∗*/*∗∗*	0.25 ± 0.13
	Calf	−0.47 ± 0.15	0.65 ± 0.17	*∗*/*∗∗*	−0.18 ± 0.13
	Foot	−0.29 ± 0.16	−0.2 ± 0.08	—	−0.23 ± 0.14

^*∗*^ *p* < 0.05 TrPs sciatica to non-TrPs sciatica group.

^*∗∗*^ *p* < 0.05 TrPs sciatica to healthy volunteers.

**Table 6:** Mean value of average temperature changes.

*T*_avr_		Sciatica patients			Healthy volunteers
TTDN phases	Subarea	Non-TrPs&DN-negative	TrPs&DN-positive	*p*	Non-TrPs&DN-negative
After DN	Thigh	−0.51 ± 0.11	0.99 ± 0.12	*∗*/*∗∗*	0.07 ± 0.12
	Calf	−0.7 ± 0.11	0.45 ± 0.11	*∗*/*∗∗*	−0.25 ± 0.10
	Foot	−0.56 ± 0.10	−0.3 ± 0.7	*∗*/*∗∗*	−0.45 ± 0.11

After observ.	Thigh	−0.38 ± 0.14	1.13 ± 0.13	*∗*/*∗∗*	0.15 ± 0.12
	Calf	−0.66 ± 0.12	0.44 ± 0.14	*∗*/*∗∗*	−0.25 ± 0.11
	Foot	−0.53 ± 0.14	−0.07 ± 0.08	*∗*/*∗∗*	−0.45 ± 0.10

^*∗*^ *p* < 0.05 TrPs sciatica to non-TrPs sciatica group.

^*∗∗*^ *p* < 0.05 TrPs sciatica to healthy volunteers.

**Table 7:** Mean value of isothermal-area below *T*_max at rest_ changes.

Isothermal-area below *T*_max at rest_ [%]		Sciatica patients			Healthy volunteers
TTDN Phases	Subarea	Non-TrPs&DN-negative	TrPs&DN-positive	*p*	Non-TrPs&DN-negative
After DN	Thigh	−27.6 ± 4.6	7.6 ± 7.8	*∗*	−2.8 ± 5.2
	Calf	−26.5 ± 5.2	3.4 ± 5.7	*∗*	−8.5 ± 4.5
	Foot	−13.9 ± 5.13	−3.9 ± 2.9	—	−9.7 ± 4.5

After observ.	Thigh	−25.07 ± 4.5	1.02 ± 7.84	*∗*	−1.42 ± 5.6
	Calf	−24.8 ± 5.7	−0.6 ± 5.3	*∗*	−10.42 ± 5.5
	Foot	−15.2 ± 5.2	−4.2 ± 2.7	—	−10.15 ± 4.2

^*∗*^ *p* < 0.05 TrPs sciatica to non-TrPs sciatica group.

^*∗∗*^ *p* < 0.05 TrPs sciatica to healthy volunteers.

**Table 8:** Mean value of isothermal-area above *T*_max⁡ at rest_  (AURP) changes.

Isothermal-area above *T*_max⁡ at rest_ [%]		Sciatica patients			Healthy volunteers
TTDN phases	Subarea	Non-TrPs&DN-negative	TrPs&DN-positive	*p*	Non-TrPs&non-DN
After DN	Thigh	0 ± 0.0	21.2 ± 2.65	*∗*/*∗∗*	1.14 ± 0.8
	Calf	0 ± 0.0	4.82 ± 1.07	*∗*/*∗∗*	0.03 ± 0.02

After observ.	Thigh	0 ± 0.0	26.4 ± 3.33	*∗*/*∗∗*	1.25 ± 0.8
	Calf	0 ± 0.0	6.63 ± 1.4	*∗*/*∗∗*	0.04 ± 0.03

^*∗*^ *p* < 0.05 TrPs sciatica to non-TrPs sciatica group.

^*∗∗*^ *p* < 0.05 TrPs sciatica to healthy volunteers.

**Table 9:** Validity and reliability of TTDN components for *T*_sk_ changes.

*T*_sk_ [°C]	TTDN phases	Area	Sensitivity [%]	Specificity [%]	Average [°C]	Min [°C]	Max [°C]	PPV [%]	ICC	*κ*
Maximum	After DN	Thigh	100	31.3	1.19	−0.2	2.6	57.7	0.622	0.702
		Calf	100	84.2	0.66	−0.8	2.1	66.7	0.422	0.786
	After observ.	Thigh	100	16.7	1.29	−0.1	2.7	54.5	0.670	0.556
		Calf	100	50.0	0.65	−0.7	1.95	85.7	0.440	0.680

Average	After DN	Thigh	100	100	0.99	0.04	1.95	100	0.400	0.880
		Calf	100	100	0.45	−0.4	1.3	100	0.511	0.902
	After observ.	Thigh	100	84.6	1.11	0.09	2.2	75.0	0.333	0.786
		Calf	100	94.4	0.44	−0.6	1.5	85.7	0.422	0.845

**Table 10:** Validity and reliability of TTDN components for isothermal-area changes.

Isothermal-area	TTDN phases	Area	Sensitivity [%]	Specificity [%]	Mean [%]	Min [%]	Max [%]	PPV [%]	ICC	*κ*
AURP (above *T*_max at rest_)	After DN	Thigh	100	100	21.2	0.6	41.8	100	0.689	0.938
		Calf	100	100	4.8	−3.5	13.1	100	0.778	0.956
	After observ.	Thigh	100	100	26.4	0.6	52.2	100	0.644	0.929
		Calf	100	100	6.6	−4.4	17.7	100	0.778	0.956

Below *T*_max at rest_	After DN	Thigh	100	0.0	7.58	−52.7	67.8	50.0	0.778	0.656
		Calf	100	20.0	3.37	−40.7	47.4	77.8	0.556	0.707
	After observ.	Thigh	100	0.0	1.02	−59.7	61.8	50.0	0.778	0.656
		Calf	100	75.0	−0.59	−41.9	40.7	87.5	0.511	0.827

Below + above *T*_max at rest_	After DN	Thigh	100	22.2	28.8	−29.3	86.9	68.2	0.578	0.696
		Calf	100	37.0	8.19	−13.6	62.8	77.8	0.578	0.782
	After observ.	Thigh	100	0.0	19.87	−5.5	45.3	48.3	0.733	0.643
		Calf	100	78.2	6.05	−12.7	57.6	79.4	0.644	0.847
